# “You can explore it more online”: a qualitative study on Australian women’s use of online health and medical information

**DOI:** 10.1186/s12913-018-3749-7

**Published:** 2018-12-03

**Authors:** Sarah Maslen, Deborah Lupton

**Affiliations:** 1 Faculty of Business, Government and Law, University of Canberra, University Drive, Bruce, ACT 2617 Australia; 2 News & Media Research Centre, University of Canberra, University Drive, Bruce, ACT 2617 Australia

**Keywords:** Digital media, Digital health, Women, Patient engagement, Feminist new materialism, Online information, Diagnosis, Australia

## Abstract

**Background:**

Previous research has demonstrated the importance of search engines, websites, online discussion groups and social media groups for women in developed countries looking for health and medical information, but few studies have focused on Australian women. The Australian Women and Digital Health Project was designed to investigate how Australian women from a range of age groups and locations used digital health technologies across the full spectrum available to them. The findings on their use of online information and decision-making in relation to seeking face-to-face medical advice are discussed in this article.

**Methods:**

Qualitative research, including focus group discussions (24 participants) and face-to-face (12 participants) and telephone (30 participants) semi-structured interviews was conducted with a total of 66 Australian women aged between 21 and 74. The focus groups and interviews were transcribed and analysed using inductive thematic analysis sensitised by a feminist new materialism theoretical standpoint. This involved identifying the dimensions of affordances, relational connections, affective forces and agential capacities in the women’s accounts.

**Results:**

All participants regularly used online sources to find health information, advice and support. We identified six key agential capacities relating to these ways in which the women enacted online health information seeking: 1) self-screening; 2) preparing for and following up a consultation; 3) selective engagement; 4) caring for others; 5) creating and sharing new information; and 6) challenging medical authority. The affordances of accessibility and convenience of online sources, relational connections between women and trusted sources (both online and offline) and between women and family members on whose behalf they sought information and affective forces such as trust, the need for reassurance and frustration and anger with deficient healthcare services contributed to these capacities.

**Conclusions:**

Women engaged in complex interactions with online information, actively and creatively using it in diverse ways in their negotiations with seeking face-to-face medical expertise. Their online practices generated a set of agential capacities that help them to assess whether they or their family members need medical attention, supplement or challenge the medical advice they have already received or generate and share their own information.

**Electronic supplementary material:**

The online version of this article (10.1186/s12913-018-3749-7) contains supplementary material, which is available to authorized users.

## Background

Since the advent of the internet and World Wide Web in the 1990s, websites and online discussion forums, and more recently, mobile applications (‘apps’) and social media platforms, have become important sources of health information for lay people. Search engines – Google Search in particular – are essential tools for finding relevant information online. These digital tools and sources contribute to the ideal of the ‘digitally engaged patient’: a lay person who actively seeks out information about health and medical issues using the internet as part of self-empowerment and health literacy efforts [1]. Despite regularly promoting this ideal [2] and studies finding that online health information tools increase patient knowledge and so their capacity for self-management [3], the medical profession can be ambivalent about encouraging patients to use digital information tools. Concerns about the power of ‘Dr Google’ to disseminate inaccurate information [4] and contribute to ‘illusory’ patient empowerment [5] or to create ‘cyberchondria’ [6–8] are regularly aired in the medical literature. Indeed, one commentator has contended that patients’ use of the internet to access health and medical information is a ‘patient autonomy problem’ that leads to patients investing more trust in the internet than in their doctors’ expertise [4]. These claims suggest concern about the contemporary status of medical authority in the context of the ever-expanding domain of health and medical information offered to lay people [9]. This article investigates how Australian women from a range of age groups and locations use online health information to support their health knowledge and decision-making and how these practices interact with face-to-face practitioner led care.

Previous research has shown that women across a range of age groups engage more highly than men with online health information sources in countries such as the United States of America (USA) [10, 11], Germany [12], France [13] and member states of the European Union [14]. The importance these sources play for pregnant women or those in the early years of motherhood has been the greatest focus of more detailed research on women’s engagements. Many women in developed countries describe constantly going online for information and peer support about pregnancy and caring for infants and young children [15–21]. It is important to note, however, that research has found that some social groups, such as low-income women in areas of the USA, rarely use the internet for health information, preferring to call upon family and friends [22, 23]. A survey of American middle-aged and older women with chronic health conditions [24] found that while 65% of them reported using the internet to find information or seek advice from others, older women were much less likely to use the internet for these purposes. Many of these women said that they preferred face-to-face engagements with healthcare providers or family and friends to seek and share information. A mixed-methods approach was adopted in a project investigating the digital health use of disadvantaged American mothers and pregnant women [25]. Findings revealed that almost all the participants had searched online for health information in the past year, but 37% did so very infrequently.

Little research thus far has been conducted on Australian women’s use of digital health. Some research has shown that, as in other developed countries, Australian pregnant women and those experiencing early motherhood continually go online to find health information for themselves and their infants [26–30]. Research on Australian women in other life stages is scant. One exception is a large survey of Australian young women (18 to 24), which found that only 43% had used the internet to search for health information. Those experiencing stigmatised conditions or symptoms (such as mental health problems) were more likely to have searched online [31].

In this article, we discuss findings from a project using qualitative methods (focus groups, face-to-face and telephone semi-structured interviews) to investigate Australian women’s use of digital health technologies, including online health and medical information sources. The Australian Women and Digital Health Project is the first study to include Australian women from a range of age groups and locations using qualitative methods, and to investigate their use of digital health technologies across the spectrum of those available to them. These methods were chosen to enable more detailed exploration of the social and biographical factors contextualising women’s use of digital health technologies than quantitative surveys are usually able to achieve.

### Theoretical approach: Feminist new materialism

The theoretical perspective we adopted when analysing research materials in this project is that of feminist new materialism, one branch of new materialisms scholarship. A range of eclectic perspectives are included under the rubric of new materialisms, they share a critique of and focus on interrogating the nature of the ‘human’, including acknowledgement of the actors (including those normatively considered to be ‘nonhuman’) that come together to configure the ‘human’ [32]. While also acknowledging social relations and interactions, feminist new materialism theory directs attention to the role played by nonhumans. It views humans and nonhumans (in this case, digital technologies) as working together to generate agential capacities, a term used in feminist materialist theory to denote the ways in which people create action and meaning with nonhuman objects [33, 34]. Barad [33, 35, 36] uses the term ‘intra-action’ to encapsulate the agential capacities generated when components of assemblages come together. This term differs from interaction by emphasising that agencies are not exchanged between one actor and another, but rather emerge with and through the entanglements of actors as they come together in assemblages and respond to and enact each other.

When focusing on the use of online health and medical technologies, from this perspective, human bodily sensations, digital technologies, other humans (for example, family members, members of online communities and medical practitioners) are involved in complex and ever-changing assemblages [37, 38]. Engagements of actors in these assemblages generate agential capacities that are always contingent and dynamic, depending on the actors that enter or leave assemblages and on time, space and place. Affordances, relational connections and affective forces can open up, or alternatively, close off agential capacities, and these intra-actions are also contingent.

A key question emerging from feminist new materialism is: what can bodies do when coming together with digital technologies (such as search engines, health websites, online discussion forums and social media groups)? Answering this question involves directing attention to the affordances of technologies and human fleshly bodies (what they allow people to do). So too, relational connections between people and between people and technologies are integral to generating agential capacities, as are affective forces – the feelings and emotions that impel action [39].Qualitative methods sensitised by this approach are able to shed light on the situated complexities of these relationships of people with technologies, including in the healthcare setting.

## Methods

### Aims, design and setting

The Australian Women and Digital Health Project was designed to investigate the following research questions: What digital technologies do women use regularly for health-related purposes, both for themselves and for any others (family members or friends)? Which do they find most and least helpful and useful? What kinds of digital health technologies would they like to see developed in the future?

The project was comprised of two separate studies. A total of 66 women participants across the two studies were involved in either interviews or focus groups about their use of digital health technologies (Table 1).

**Table 1:** Participant details

Study 1 (Canberra)	• community forum (2 groups): 11 participants, age range 28—65 • face-to-face interviews: 12 participants, age range 21—63 • focus groups (3 groups): 13 participants, age range 25—58
Study 2 (Australia-wide)	• telephone interviews: 30 participants, age range 22—73

The same semi-structured interview schedule was used with all participants. The participants were asked which technologies they used and found valuable or useful for their everyday engagements and practices related to health and wellbeing. The participants were also asked to reflect on what type of digital technology they would like to see invented that would fit their needs: in effect, to articulate their own imaginaries about the potential of digital health. These questions provided the basis of the interviews and group discussions, but interviewers also probed participants for further comments and explanations of their responses, allowing for free-ranging conversations. The interview protocol was developed for the purposes of this study and had not been used previously (see Additional file 1).

### Ethics approval

Ethics approval to conduct this research was granted by the University of Canberra’s Human Research Ethics Committee. All participants were provided with project information and gave their written consent to participate. They were all given pseudonyms to protect their anonymity.

### Participant characteristics

*Study 1* involved three sets of women living in Canberra, totalling 36 participants. The first set included a total of eleven women who attended an initial community forum which was advertised among women’s community health groups by Women’s Centre for Health Matters (WCHM), a community-based not-for-profit organisation that works in Canberra and surrounding regions to improve women’s health. The participants who attended the forum were divided into two focus groups, one of which was led by the second author and the other by a staff member from the community centre. Their ages ranged from 28 to 65 years. Following this forum, another twelve participants (aged from 21 to 63) were recruited to take part in individual face-to-face interviews. Three further focus groups with a total of 13 women were also conducted. One focus group consisted of six women with young children (aged from 25 to 33), the second included four women with young children who were part of a support group for mothers living with mental health conditions (aged from 25 to 30) and the third focus group included three women aged in their mid-to-late 50s. Of the total of 36 women involved across these Canberra participant groups, 28 identified their ancestry as Anglo-Celtic and eight as Asian. Twenty-two participants reported university-level education, while fourteen had high school or technical qualifications.

These interviews and focus groups were conducted by two research assistants employed on the project. The participants were recruited using WCHM’s networks, personal contacts, advertising on relevant Facebook pages (such as those for mothers, people with disabilities and women’s fitness and sporting groups in Canberra) and posters in public places around the city. They took place in a range of locations, including places where the focus group participants usually met, homes and cafes.

*Study 2* involved telephone interviews with 30 women living in various locations around Australia. A market research company was commissioned to recruit the participants and conduct the interviews. Participant information and consent were provided online before the interviews were conducted. This group of participants were recruited using sub-quotas based on age, to ensure a good spread of ages: 10 aged 18 to 40, 10 aged 41 to 60, 10 aged 61 and over. These participants ranged in age from 22 to 74. Two-thirds lived in major cities or towns, one third lived in rural Australia. Twenty participants lived in the state of New South Wales, four in Queensland, five in Victoria and one in Western Australia. Twenty-four participants described themselves as having Anglo-Celtic ancestry, one as Western European, two as Southern European, two as Asian and one as Middle Eastern. Of this group, 14 reported university qualifications, and the remaining 16 participants had high school or technical qualifications.

### Analysis

All the group discussions were audio-taped and transcribed by a professional transcription company. The authors worked together to analyse the transcripts using inductive thematic analysis [40] informed by feminist new materialism. This involved identifying recurring themes within and across each group discussion by reading and re-reading the transcripts, locating the places where the participants talked about the digital information that they accessed from online media and considering the following dimensions: the affordances of the human bodies and technologies involved; relational connections; affective forces; and the agential capacities generated when these dimensions intra-acted. For the purposes of the analysis presented in this article, we kept these dimensions in mind when considering the participants’ accounts of how and why they searched for health information online. Each author conducted initial analysis separately and then combined their insights when co-authoring the final analysis.

Our analysis is presented by first outlining the key affordances offered by online media as articulated in the participants’ accounts, followed by discussion of five major agential capacities generated by participants’ enactments of health information-seeking online. Verbatim quotations from the discussions were chosen to provide support for the thematic analysis.

## Results

### Key affordances

All of the women who participated in our research, regardless of their age, ethnicity or educational background, reported regularly searching online (typically described as ‘googling’ or using ‘Dr Google’) for health information, advice and support. For most of the participants, searching online was their first port of call. The majority of participants noted that one of the benefits of online sources is that they could search for information or seek peer advice in online support forums. Making an appointment with a medical professional meant taking time to find an appointment that would suit them and then attending.

I think because it’s so easy, the computer’s in the room. I can give you an example: when my daughter was pregnant she had high blood pressure. Straight away I would go to Google, type high blood pressure and pregnancy and try and get more information that way … It’s definitely more accessible, trying to get a doctor’s appointment obviously can be time consuming and you may not get the appointment that you need. (Leanne, 55, high school education, Anglo-Australian)

The women noted that medical services are not always available when they are required, either because it is a weekend or because people live in a location where services are stretched. In these cases, online sources offer an alternative. For example, Paula (36, university degree, Anglo-Australian) lives in a small town, and has difficulty getting medical appointments when she needs them. She has a serious chronic mental health condition that requires regular medical attention, but the strain on medical services in her area means that she is forced to go online instead.

It can be up to a week before people can get in to see doctors here, so if you want information, like, then and there, or sooner than a week, it’s easier to go online and try and source it yourself.

These initial accounts, therefore, highlight the key affordances offered by online technologies: accessibility and convenience. Further analysis of the participants’ accounts found that while online searching was practised by all the participants, they engaged in complex interactions with online information, using it in diverse ways in their negotiations with seeking face-to-face medical expertise. We identified six main agential capacities: 1) self-screening; 2) preparing for and following up a consultation; 3) selective engagement; 4) caring for others; 5) creating and sharing new information; and 6) challenging medical authority. These capacities are not exclusive of each other: women may take up a combination of them in their everyday information-seeking practices, depending on the context. They are discussed in detail below.

### Self-screening

Many of the participants’ health-related online searches focused on investigating their symptoms as an initial screening tool. Women may then go to make the decision to seek a consultation with a doctor, either because their self-assessment suggests their condition may be serious enough, they want reassurance, or if symptoms persist. It was evident from some women’s accounts that they viewed the practitioner consultation as largely confirming their self-diagnosis after accessing relevant information online. This confirmation is critical because the practitioner has diagnostic expertise, as well as the authority to prescribe medication or refer patients to specialists for further treatment.

[Searching online] sort of assists me in planning what I should do – whether it’s something I should go to the doctor about or whether it’s something that I can handle myself. It’s more a diagnostic tool that I use. I know you shouldn’t, but it’s what I do use it for. A diagnostic assistant. (Margaret, 73 years, high school education, Anglo-Australian)

As these women’s responses demonstrate, people are aware that the medical profession often frowns on patients using ‘Dr Google’ (as Margaret says, ‘I know you shouldn’t’), but they are also conscious of the importance of not seeking medical attention if it is unwarranted. As some participants noted, it depends on the seriousness of the health condition to what extent they sought further medical advice. Conducting a search online is a way of determining whether their or a family member’s symptoms are ‘serious enough’, as Sharon (59, high school education, Anglo-Australian) notes, to make the effort to take further steps involving consulting a doctor.

### Preparing for and following up consultations

Even when women plan to present at a general practitioner, they can see researching their symptoms online in advance as valuable. This research supports their health understanding so they can more readily engage in the advice from practitioners. Katrina (38 years, university degree, Anglo-Australian) lives with ulcerative colitis, a chronic digestive disorder, and consults regularly with her general practitioner. She noted that:

I find that if I do the web stuff before I go to the GP I feel I’m a bit more prepared to hear what they’re probably going to tell me and get reassurance from them. So – I mean, I kind of think I’m a fairly health literate person. So in my case I’m quite happy to look up things on the internet and be like well, that’s from a relatively good source, that's from a dodgy source or whatever. But at the end of the day I don’t have a degree – a medical degree anyway. So I would like to go and see my doctor or my specialist just to make sure that what I’m thinking is actually the same thing.

An initial online search can also be a way of arriving at a medical consultation with a clear plan of what to ask of the doctor. This was evident from the account of Marilyn (67 years, high school education, Anglo-Australian), who explained that she uses online information as a platform to become informed and advocate for treatment:

Anything that shows up for me I normally google it and look through it, see if I can get any clues to what it is. And then when I go and see the doctor about it, I demand to have the sort of care that I’m supposed to get instead of being patted on the head and sent away.

Women may also go back to online sources having consulted a practitioner, as a way of further looking for details about their diagnosis and self-management of their condition. As Marie (73, university degree, Anglo-Australian) commented:

Well, you can explore it more online. The doctor will treat it, usually treat the symptoms but doesn’t go into any sort of genetic predispositions or doesn’t tell me about what the medication does or any possible side effects. So I can research that properly online, myself. If I’m having symptoms I can find out if something is wrong, and if it’s really bad I wouldn’t self-medicate, I would go back to the doctor and say, “Look, this isn’t my imagination, it’s this”.

### Selective engagement

While the participants were actively managing their own health using different information sources, they are aware of the limits of their health knowledge. Several women commented about the risk of finding information online that would cause unfounded anxiety. They viewed visiting the doctor as the way to avoid this. Emily (25 years, university degree, South-East Asian) noted that while she would go to the internet first for health information, she considered doctors to be the most important source of information:

Because Google could tell you that you’re dying or have brain cancer but really you just have a bad flu. So I just tend to trust that the doctor’s got a bit more knowledge than me.

The majority of participants expressed caution about the accuracy and validity of the health and medical information they found online and, in some cases, expressed difficulties in knowing how to assess this information. Concerns about online health information mean that evaluation of information quality is important. Most women had some kind of system for evaluating quality, including a strategy of also seeking advice from a practitioner or taking advice from medical practitioners about where to find quality information. Some women looked for government health department websites, those run by high-profile non-government organisations or well-known medical websites such as WebMD. Others preferred peer-to-peer resources in online forums or social media groups, perceiving these to be less driven by an agenda.

Several women expressed concerns about sources they identified as profit-driven rather than oriented towards patients’ best interests. As Houda (45, university degree, Middle Eastern) commented of online forums she looked at:

You get different perspectives from different people, I think they are real people, I don’t think they have any agenda behind what they’re saying. If you go on to a health website you think, “Ok, who’s funding it? What pharmaceutical company?” Do you know what I mean? I think they [the people contributing to online forums] are a bit more honest than websites.

Some women were selective about the kinds of information they would principally seek online. For instance, they may look for general health information such as that on diet from online sources but seek medical attention for topics they deem to be ‘medical’*.* Women also valued local sources of information because they considered them to be more relevant to their own circumstances. A common strategy of evaluating the validity of an internet source articulated by participants was determining whether it was Australian:

I mainly go by whether it’s an Australian-based site. If I can’t tell by the link that it takes me to, I look for contact details to see if it’s an Australian-based site. So I tend to believe Australian-based information because it’s more relevant than what another country’s health information would be. (Rosa, 36 years, university degree, Anglo-Australian)

### Caring for others

Women’s practices of using online search tools to support health assessments and find information also extended to family members. In engaging in this practice, they were performing the role of carer, taking responsibility for seeking information on behalf of their family member. As one woman explained, googling for information allowed her to find health information for herself and her children across a diverse range of topics:

You can get results for what you’re searching for whether it be something related to me specifically, like my own workout or food or my own wellbeing like probiotics to take and that kind of thing, or it’s something related to my daughter like baby food or that kind of thing. Or it’s just something for my older child: you know, websites specific to her disability or googling about medications or conditions, because I pretty much do all the health sort of stuff for the whole family. (Hannah, 34 years, university degree, Anglo-Australian)

It is not only young children for whom women were frequently seeking online information. They also searched on behalf of their adult children, partners and elderly parents. Another example is Susan (56 years, university degree, Anglo-Australian), who has an adult son with Asperger’s syndrome and a husband living with diabetes and a heart condition. She said that she frequently goes online to find information related to their conditions that she then shares with her son and husband. Sandra (55 years, high school education, Anglo-Australian) supported her mother-in-law’s health by searching online:

My mother-in-law had shingles, and so straight away I’d go to Google and try to find out what the symptoms are, you know, how to cure it, what’s the best way to treat it, that sort of thing.

### Creating and sharing new information

For women living with or caring for children with chronic health conditions in particular, online sources were often used as a form of lay creation and sharing of knowledge. These women used discussion forums and social media groups to help them find a label for conditions that previous medical consultations were unable to identify, thus achieving their own diagnoses. Once a diagnosis was achieved, women with a chronic health condition used the internet to find support communities or in some cases, create their own. These communities were used to keep abreast of existing knowledge (treatments, tests) on their condition.

Louisa (30 years, university education, Anglo-Australian) is one such example. She lives with several chronic pain conditions, to the point that she is too unwell to work, although she studies part-time at university. She said:

I self-diagnose a lot because quite honestly, I know my body better than they do. I have two doctors who basically go, “Well I think it could be this, do you want to go home and google it and tell me what you think?” They trust me with Google enough to do that. Just the other day … my doctor said to me, “I want you to try this new medication, but you’ve got to go home and read about it first and see if you decide if it’s what you want to try or not.” So sometimes I go, no, well I think it’s going to have these side effects or like I read this about it and I’m not comfortable taking it, or I think, no, no, it sounds really good, I’ll give it a go.

From Louisa’s perspective, online information helps make sense of her embodied, self-knowledge towards a diagnosis. It also provides information about treatment options and how they might be physically experienced. She is then able to be actively engaged in the discussion with her practitioner over the course of action.

These practices of seeking peer information and support can extend from women’s own health to that of family members with chronic conditions and disabilities. However, it does not necessarily exclude recourse to the authority of medical expertise. For example, Rachel (38, technical certificate, Anglo-Australian) is interested in alternative health. She explained that she uses many Facebook groups to find information on this topic. Accessing these groups gives her fast answers to her health questions, but through selecting to ask questions in different forums she also ensures her guidance is relevant to her values. While Rachel is very actively involved in multiple online peer communities in this way, she is also conscious of their limits and does not always use this information source if she feels she needs a face-to-face medical consultation:

I’ve got spots on my hand, it could be dermatitis, it could be hand foot and mouth, it could be a rash, like you know, it’s huge. Whereas if the doctor looks at it “Ah yes, you have a rash.” It’s easy for them to see it straight away. But if you’re googling stuff, or you’re using the internet, or even social media for that matter, they don’t know the full story and they can’t see what you’re talking about – it’s just their opinion. But if you go to the doctor, the doctor has studied for this and they know what they’re looking for and they know how to deal with it.

Similarly, Susan (56, university education, Anglo-Australian) said that she thinks doctors are an important part of healthcare for her son and husband, but also notes the limits of their knowledge, particularly in new developments: ‘quite often I’ll do research to see if there’s anything new coming out’. The online information Susan finds is then shared with her doctor, who is able to help her assess its validity and value. She describes a partnership between herself and her doctor.

### Challenging medical authority

For a small number of women, online information had superseded face-to-face medical expertise*.* They now relied on doctors mostly to generate an official diagnosis and prescribe tests. One example is Justine (38, high school education, Anglo-Australian). Like Louisa, Justine lives with chronic pain related to multiple conditions. She commented that she has found online research very helpful due to inadequacies in her treatment with practitioners, but her doctors have discouraged her from information seeking in this way. She said that getting to the bottom of these issues resulted directly from her own research and pushing practitioners for tests to investigate.

[Doctors] always say don’t use Dr Google, but you know what, it’s helped me immensely. I have no high regard for the medical [profession]. Yeah, but I still have issues where I have to rely on them … I don’t go in thinking well you must be an amazing person because you have a degree.

Another example is Megan, who is 48, university educated and Anglo-Australian. Megan lives with lipoedema, a chronic condition mostly affecting women that causes abnormal building of fatty tissue in the limbs and buttocks and is often mistaken for obesity. Her dissatisfaction with the mainstream medical engagement with her condition drives her own information seeking/sharing. Megan does a lot of online research, and also very actively participates in support groups on Facebook, both sharing and receiving information on her condition in the form of links to research, papers, journal and media articles.

After I received my diagnosis, I looked at what I could find online, and because there just isn’t enough medical support for it in Australia. I realised that there really wasn’t a lot out there to help me. I quit my job, and basically decided to turn myself into a human guinea pig to see if I could find my own treatment based on all the different research papers that I’d read and that sort of thing. So, I did. For six months, I read everything that I could find online, and worked out some wide-ranging treatment protocols. I got on top of the disease.

The group Megan started on Facebook now has 1500 members. Megan is able to share all the information she has curated through her online searches on her Facebook community, thereby establishing herself as an authority in her own right. She said that she only goes to a doctor now when she needs referrals, and in these appointments, she requests what she’s after rather than asks for advice. Megan observed that while her original general practitioner was willing to work with her as a partner, other doctors have found her ‘empowered patient’ approach more threatening:

My regular GP was very helpful and put me on a chronic disease management plan, which gave me the five allied health appointments. But then she left town and her replacement was very intimidated by a patient that knew more about her disease than she did. So I just stopped going. So now I only visit GPs if I need something like referrals, and I basically go in with all my research, and tell them what I need the referral for. They get shitty about that.

Megan is an example of a ‘digitally engaged patient’ who has crossed the line for many of the doctors she consults. She has become too empowered and too challenging of traditional models of medical authority. As a result, she experiences antagonism from most of the general practitioners she has consulted.

## Discussion

Our findings contribute to and extend previous research on women’s use of online health information sources in developed countries by focusing on more detail on how they access and use their sources in relation to medical consultations. Regardless of their age or education level, our participants were even more highly engaged in seeking health information online compared with the participants in studies based in the USA [10, 11, 22–24], the European Union, Germany [12] and France [13]. This finding may be partly due to the very high use of the internet and smartphones by Australians more generally compared with other populations [41]. Given that our research was conducted more recently than previous studies, our findings may also reflect the growing importance of online sources to lay people as more resources have become available over time.

In identifying the agential capacities generated when our participants engaged with health and medical information online, our research findings provide a more nuanced perspective than is often advanced from a health services viewpoint. The women enacted complex interactions with online information sources, using them in diverse ways in their negotiations with seeking face-to-face medical expertise. The affordances of accessibility and convenience of online sources were integral to the agential capacities generated. So too were the relational connections between women and trusted sources (both online and offline) and between women and family members. Affective forces such as trust, the need for reassurance, care for others, the desire to feel more in control and frustration and anger with deficient healthcare services contributed to these capacities. Notably, many of the participants were performing an important caring role in regularly supporting the health of their family members by seeking online information and advice on their behalf. In doing so, they were responding to the sociocultural norm that women should engage in this kind of informal healthcare labour for others [42, 43].

We found little evidence of a ‘patient autonomy problem’ in which patients are investing their trust in online sources over medical practitioner expertise. The efficacy of digital health tools to support self-diagnosis ultimately relies on patients’ own embodied knowledge and their capacity to articulate their symptoms using medical language. While they are expert in knowledge of their own bodies, they do not share the language of a health practitioner or clinical and diagnostic expertise. Some women were using these sources to be an informed patient able to actively participate in their healthcare. Others used online sources principally due to their accessibility, even if they ultimately decided to go to the doctor. In these instances, online information sources were used as a self-screening tool ahead of the anticipated consultation, helping them prepare for their visit to the doctor. They wanted to avoid seeking unnecessary medical advice, expending time and money in doing so and possibly taking up the doctor’s attention when it was not needed. At the more extreme end of patient engagement with online information, some women with chronic illnesses in particular were directly challenging medical expertise and creating their own communities online as an alternative. They were in the minority, however, and were responding in these ways because they had experienced difficulties in being taken seriously by the medical practitioners whose help they had sought or because orthodox medical expertise had not provided solutions to their health conditions.

### Limitations and directions for future research

While our research was able to generate a number of important insights into Australian women’s use of online sources for health and medical information, the participants were not recruited randomly and the findings are not necessarily generalisable. While some women living in rural regions and those without university education and from non-Anglo-Australian backgrounds were included in our project, we could find little differences between women from these groups and other participants. In Australia, these groups are more likely to experience greater ill-health, lack of access to digital technologies and lower levels of digital and information-seeking skills [44, 45]. The main differences in information-seeking practices we noted were between women with chronic illnesses and those who did not live with such conditions. Further indepth research including more participants, both women and men, with chronic illnesses or disabilities and those from socioeconomically disadvantaged groups and rural and remote locations would build on our findings.

## Conclusions

Australian women’s use of digital technologies for health and medical information is diverse, generating a set of agential capacities that help them to assess whether they or their family members need medical attention, supplement or challenge the medical advice they have already received or generate and share their own information. These agential capacities are generated across a range of contexts that relate to the severity of symptoms or illness under investigation and whether they are considered unusual, chronic or have not yet been effectively diagnosed by medical practitioners. It was not simply a matter of self-knowledge of one’s body or information from the internet being privileged over medical expertise. All these diverse forms of knowledge and sense-making worked together to better configure understanding of the women’s health status (and those of their family members).

Above all, our findings point to the high value that women place on being able to access online resources readily, as well as the complexities of the rationales and practices involved. They were conforming to the ideal of the digitally engaged patient, working to learn about their bodies and health, preparing for medical consultations and avoiding seeking medical attention if it was not required. Far from the often very simplistic and paternalistic views on the digitally engaged patient presented in the medical literature, our findings demonstrate that trust in medical expertise is in most cases not foregone by people going online. Instead, our participants were engaging actively, creatively and critically with online information, using it in a number of different ways to complement rather than supplement medical advice.

## Additional file

Additional file 1: Australian Women and Digital Health Project Interview and Focus Group Questions. Provides the questions developed for our study to facilitate the focus groups and one-on-one interviews. (DOCX 13 kb)

## Abbreviations

USA: United States of America
WCHM: Women’s Centre for Health Matters
